# [Expression of Concern] Downregulation of microRNA-183-5p inhibits the proliferation and invasion of colorectal cancer cells by inactivating the reticulocalbin-2/Wnt/β-catenin signaling pathway

**DOI:** 10.3892/mmr.2025.13659

**Published:** 2025-08-20

**Authors:** Gang Wang, Jingrong Zhou, Feng Lu, Lei Qiu, Liang Xu, Xiuwei Yang, Yongchang Miao

**Affiliations:** Department of Gastrointestinal Surgery, The Second People's Hospital of Lianyungang, Haizhou, Jiangsu 310000, P.R. China

Mol Med Rep 19: 4475–4483, 2019; DOI: 10.3892/mmr.2019.10059

Following the publication of this paper, it was drawn to the Editor's attention by a concerned reader that, regarding the Transwell assay experiments shown in Figs. 3 and 4, the ‘Invasion/NC’ panel in Fig. 3H appeared to contain an overlapping section of data with both the ‘Invasion/NC’ 'and the ‘Invasion/'miR-183-5p mimic+RCN2 siRNA’ panels in Fig. 4C, such that data which were intended to show the results from differently performed experiments had apparently been derived from the same original sources. The authors were contacted by the Editorial Office to offer an explanation for these apparent anomalies in the presentation of the data in this paper; however, up to this time, no response from them has been forthcoming. Owing to the fact that the Editorial Office has been made aware of potential issues surrounding the scientific integrity of this paper, we are issuing an Expression of Concern to notify readers of this potential problem while the Editorial Office continues to investigate this matter further.

